# Supplementary material for: Landscape of FLT3 Variations Associated with Structural and Functional Impact on Acute Myeloid Leukemia: A Computational Study
Source: Int J Mol Sci. 2024 Mar 18;25(6):3419. doi: 10.3390/ijms25063419 (PMC10970727; doi:10.3390/ijms25063419)
Supplement: Supplementary file 1 [file ijms-25-03419-s001.zip › ijms-2877317-supplementary.pdf]

Supplementary Table S1: All variations listed in ClinVar for FLT3 having missense amino acid change – 86 single nucleotide variation and 3 deletion (https://www.ncbi.nlm.nih.gov/clinvar; accessed 31 January, 2024).

	**Protein change**	**Condition (s)**	**Clinical Significance**
1.	E991K	not specified	-
2.	A988P	not provided	benign
3.	P986L	not specified	-
4.	R973Q	not provided	Likely benign
5.	S963L	not provided	Likely benign
6.	R961H	Inborn genetic diseases	Likely benign
7.	R961C	Inborn genetic diseases	Uncertain significance
8.	A953V	not specified	-
9.	F906L	Inborn genetic diseases	Uncertain significance
10.	I881T	Inborn genetic diseases	Uncertain significance
11.	W872C	Inborn genetic diseases	Uncertain significance
12.	V852I	not provided	Likely benign
13.	R845G	AML	Likely pathogenic
14.	Y842C	AML	Likely pathogenic
15.	Y842H	AML	Likely pathogenic
16.	N841K	AML	Likely pathogenic
17.	N841H	AML	Likely pathogenic
18.	D839G	AML	Likely pathogenic
19.	I836del	AML	Likely pathogenic
20.	I836M	AML	Pathogenic
21.	I836S	AML	Likely pathogenic
22.	I836F	AML	Likely pathogenic
23.	I836V	AML	Likely pathogenic
24.	I836L	AML	Pathogenic
25.	D835E	AML	Pathogenic
26.	D835del	AML	Pathogenic/Likely pathogenic
27.	D835F	AML	Likely pathogenic
28.	D835A	AML	Pathogenic
29.	D835V	AML, ALL	Pathogenic
30.	D835Y	AML, ALL	Pathogenic
31.	D835N	AML	Pathogenic
32.	D835H	AML	Pathogenic
33.	C828R	Inborn genetic diseases	Uncertain significance
34.	M737I	AML	not provided
35.	K709T	Inborn genetic diseases	Uncertain significance
36.	F691L	AML	Likely pathogenic
37.	I687F	AML	Likely pathogenic
38.	S684P	not specified	-
39.	N676K	AML	Likely pathogenic
40.	N676D	AML	Likely pathogenic
41.	E672K	Inborn genetic diseases	Uncertain significance
42.	K663Q	AML	Likely pathogenic
43.	D651G	AML	Likely pathogenic
44.	G619C	AML	Likely pathogenic
45.	F594L	AML	Likely pathogenic
46.	D593del	ALL	Pathogenic
47.	V592A	AML	Likely pathogenic
48.	Y591C	AML	Likely pathogenic
49.	Y591D	AML	Likely pathogenic
50.	V579A	AML	Likely pathogenic

51.	Y572C	AML	Likely pathogenic
52.	Y572H	Multiple myeloma	Likely pathogenic
53.	V557I	not provided	Benign
54.	N541S	Inborn genetic diseases	Likely benign
55.	I507V	not provided	Likely benign
56.	S504R	Inborn genetic diseases	Uncertain significance
57.	G493V	Inborn genetic diseases	Uncertain significance
58.	T475P	not provided	Likely benign
59.	S461F	Inborn genetic diseases	Uncertain significance
60.	M430I	Inborn genetic diseases	Uncertain significance
61.	K429T	Inborn genetic diseases	Uncertain significance
62.	I417L	not provided	Benign
63.	Q394R	not specified	not provided
64.	I361T	Inborn genetic diseases	Uncertain significance
65.	D358V	not provided	Benign
66.	S356N	Inborn genetic diseases	Uncertain significance
67.	D324N	not specified	Likely benign
68.	V317E	Inborn genetic diseases	Uncertain significance
69.	N306H	Inborn genetic diseases	Uncertain significance
70.	G295D	Inborn genetic diseases	Uncertain significance
71.	A291P	Inborn genetic diseases	Uncertain significance
72.	G282R	not specified	not provided
73.	F281L	Inborn genetic diseases	Uncertain significance
74.	N278T	not specified	not provided
75.	V275I	Inborn genetic diseases	Uncertain significance
76.	L262F	not specified	not provided
77.	L257W	Inborn genetic diseases	Uncertain significance
78.	R243K	not provided	Likely benign
79.	T227M	not provided	Benign
80.	Q202R	not specified	not provided
81.	V194M	not provided	Benign
82.	K175R	Inborn genetic diseases	Uncertain significance
83.	N151S	not specified	not provided
84.	P116R	not provided	Likely benign
85.	D84G	Inborn genetic diseases	Uncertain significance
86.	G64R	not specified	not provided
87.	I38N	Inborn genetic diseases	Uncertain significance
88.	V16L	not provided	Likely benign
89.	D7G	not provided	Benign
